# A core outcome set for clinical trials of first- and second-degree perineal tears prevention and treatment: a study protocol for a systematic review and a Delphi survey

**DOI:** 10.1186/s13063-021-05820-6

**Published:** 2021-11-25

**Authors:** Lin Xiao, Lei Shi, Suting Liu, Yuanyuan Luo, Jinhui Tian, Lili Zhang

**Affiliations:** 1 Evidence-Based Nursing and Midwifery Practice Center, School of Nursing, Southern Medical University, Guangzhou, 510515 China; 2 Evidence-Based Medicine Center, School of Basic Medical Sciences, Lanzhou University, Lanzhou, 730000 China

**Keywords:** Consensus, Core outcome set, Delphi, First-degree perineal tear, Second-degree perineal tear

## Abstract

**Background:**

Perineal tear is a common consequence of vaginal births affecting females globally. Even mild perineal tears could cause short- and long-term complications for females. Though many studies of interventions to prevent or treat perineal tears to minimize the consequences have been conducted, there is a significant heterogeneity in the outcomes measured and reported in existing studies, which makes meaningful comparison difficult and makes the generalizability to clinical practice challenging. Developing a core outcome set (COS) could solve these methodological concerns. In this paper, we report a protocol to develop a COS for clinical trials of mild perineal tears, which shall assist in establishing the evidence base and implementation of effective measures to reduce the incidence and minimize the consequences of mild perineal tears.

**Methods/design:**

The development of this COS will be guided by a study advisory group composed of obstetricians, midwives, nursing managers, service users, and methodologists. This study will include four stages: (1) a systematic research of the literature to identify outcomes reported in prior studies, (2) a semi-structured interview with key stakeholders to collect their opinions on important outcomes, (3) a panel of experts will be invited to conduct a three-round Delphi survey to prioritize these outcomes, and (4) a consensus meeting with key stakeholders to determine the list of outcomes included in the final COS.

**Discussion:**

The development of this COS will provide international standards for the outcomes to be collected and reported in all clinical trials and audits of practice, which involve prevention and treatment of first- and second-degree perineal tears for women with vaginal delivery. This will facilitate comparing and contrasting of studies and allow for combining of appropriate studies with the ultimate goal of improved perineal care for women choosing vaginal delivery.

**Trial registration:**

This study was registered in the database of Core Outcome Measures in Effectiveness Trials (COMET) on June11th, 2021 (https://comet-initiative.org/Studies/Details/1884).

## Background

Perineal tear is a distressing symptom affecting females globally. Previous studies reported more than 80% of women sustain some degree of perineal tear during childbirth [1, 2], which is one of the most common complications of vaginal delivery, and it is associated with short- and long-term complications such as persistent pain [3], dyspareunia [4], and urinary and anal incontinence [5, 6]. Severe perineal tears (third and fourth degrees) cause a more serious impact on women, which could even affect women’s psychological well-being and family relationships [7]. Most previous research focused on severe perineal tears, and less attention has been paid on mild perineal tears (first and second degrees). However, a large cohort study found second-degree tears alone may impair sexual function [8] and increase the risk of future pelvic organ prolapse [9]. Moreover, the incidence of mild perineal tears is very high. It has been reported that the incidence rate of second-degree perineal tears is 35.1–78.3% among primiparous women and 34.8–39.6% among multiparous women [2, 10, 11]. Therefore, the mild perineal tears should also deserve attention. Currently, researchers have carried out a large number of studies on the risk factors, diagnosis, and prevention of perineal tears to provide support for perineal tear prevention. Various interventions such as antenatal perineal massage [12], warm compresses [13], hands on the perineum [13], and Ritgen’s maneuver [13], to reduce perineal trauma, have been studied, and positive benefits associated with some interventions have been reported. However, the outcomes of the existing studies reported are numerous and highly variable, and the techniques for measuring many outcomes are also poorly defined, making it difficult to compare studies. As a result, no totally agreed schemes for the prevention of mild perineal tears have been made. In addition, 60 to 70% of perineal tears need to be repaired [14] and should receive care following repair. Similarly, the great inconsistency of reported outcomes of related primary studies limits the ability to synthesize the results of individual studies in a meta-analysis [15], which has caused great obstacles for best evidence-based practice in this clinical area. Another problem is the outcome reporting bias, where a large number of outcomes are measured but only those that show interesting or positive results are reported, resulting in a biased view of the results of a trial. Besides, it is also unclear how relevant, if at all, these outcomes are to service users. In short, the important issue to be solved is how to select relevant and important outcomes to key stakeholders if the study findings are to influence policy and practice, finally to facilitate the development of high-quality care.

One solution to these problems is to develop a COS, which is a minimum dataset that trials for a given condition should report [16]. It represents a minimum dataset that should be collected and reported but does not restrict researchers from adding additional outcomes at their discretion. The development of a COS across multiple disciplines is supported by the Core Outcome Measures in Effectiveness Trials (COMET) initiative, which brings together interested researchers and minimizes duplication of work [16, 17]. Currently, a COS for severe perineal tear prevention and treatment is under planning but has not yet been established [18]. Our study aims to present a protocol for a study to develop a COS for the prevention and treatment of mild perineal tears, so as to provide a comprehensive outcome set for different degrees of perineal tears.

## Scope, aim, and objectives

### Aim

The aim of this study is to develop an initial COS suitable for studies assessing the effectiveness of prevention interventions for mild perineal tears and assessing the efficacy of any treatment strategies to manage mild perineal tears.

### Scope

The COS is designed for use in both research and routine clinical care, in any health care system with midwifery qualifications. It should cover all women of childbearing age and should apply to all interventions for prevention or treatment of mild perineal tears in women with vaginal delivery.

### Study objectives

The specific study objectives are as follows:

1. Complete a systematic literature review to identify the outcomes reported in prior studies and conduct semi-structure interviews with key stakeholders to collect their opinions on the outcomes of prevention and treatment of mild perineal tears for women with vaginal delivery.
2. Prioritize these outcomes from the perspective of key stakeholders, including women experiencing mild perineal tears, midwives, obstetricians, nursing managers, and researchers with expertise in perineal care, using a Delphi survey and consensus meeting.

## Methods/design

This study has been registered on the COMET website (No.1884 )[19]. We will develop the COS based on the general guidelines of the COMET handbook [20], COS-STAD [21], and consensus-based standards for the selection of health measurement instruments (COSMIN) [22]. It will include four stages: (1) a systematic review of the literature to identify outcomes reported in prior studies, meanwhile, a study advisory group (SAG), composed of key stakeholders including obstetricians, senior midwives, senior nursing managers, service users, and methodologists, will be set up to evaluate the identified outcomes; (2) a semi-structured interview with key stakeholders to collect their opinions on additional outcomes which have been left off the checklist; (3) a panel of experts will be invited to conduct a three-round Delphi survey to prioritize these outcomes; and (4) a consensus meeting with key stakeholders to determine the list of outcomes included in the final COS.

### Stage 1: A systematic research of outcomes in published studies

According to the COS development process in the COMET handbook [20], it is recommended that potential relevant outcomes are identified from existing work to inform the consensus process. A systematic research of the literature is advantageous because it can efficiently identify an inclusive list of outcomes being reported by researchers in a given area, which shall be one of the main resources to constitute the content for the Delphi study.

#### Search question

What are the outcomes reported in studies assessing the effectiveness of prevention or treatment of mild perineal tears for women with vaginal delivery in any health care system with midwifery qualifications?

#### Literature search

Using a comprehensive search strategy, the following databases will be searched for relevant studies: PubMed, Embase, Cochrane Library, CINAHL, Wanfang database, CNKI, and Chinese BioMedical Database. ClinicalTrials.gov will also be searched for relevant, ongoing trials. Key terms used to guide the search will include “perineal tear,” “perineal trauma,” “perineal laceration,” “perineal injury,” “vaginal tear,” “first-degree tear,” and “second-degree tear” combined as appropriate using the Boolean operands “OR” and “AND.” The time limits applied to each of the databases are from the establishment of the database to the time of retrieval. The reference lists of all relevant studies will be searched for additional relevant studies not retrieved from the electronic database search. Language restrictions will not be applied to the search strategy; however, the selection of relevant articles will be restricted to English or Chinese language publications. Searching all languages will enable us to identify the extent of potentially eligible additional studies that will not be included and consider if this presents a source of language bias.

#### Types of studies

Both secondary studies (systematic reviews, meta-analysis) and original studies (randomized controlled trials, cohort studies, case-control studies) comparing women who did and did not receive prevention or treatment interventions of perineal tears will be included in our literature review. In line with prior work in this area, we will exclude review reports and reports of conference proceedings or abstracts when there is no complete description of the trial or study. If a study focuses on all subtypes of perineal tears (both mild and severe perineal tears), the full text will be read to decide whether to include it.

#### Types of interventions

The interventions include prevention and treatment strategies, including the following as a sole intervention or in combination. Prevention interventions include pelvic floor muscle training, perineal massage, delivery posture, warm perineal compression, manual perineal protection, pushing**,** episiotomy, instrumental delivery, and epidural analgesia. Treatment interventions include suture materials (e.g., absorbable synthetic sutures (fasting absorbable, standard absorbable), catgut (plain, chromic, and glycerol impregnated)), suture techniques (e.g., conventional suturing, perineal skin suturing, continuous suturing, interrupted suturing, overlapping repair, end-to-end repair, two-stage repair, three-stage repair, inverted interrupted skin sutures, continuous subcuticular suture, continuous non-locking suture, non-suturing), repair personnel, repair time, and postoperative management (e.g., pain relief, infection prevention, wound care).

#### Types of participants

All women of childbearing age, pregnant women, or women experiencing mild perineal tears, regardless of sex, age, or race, will be included. The participants for prevention interventions will be pregnant women, pregnant women about to give birth, or women in labor with vaginal delivery. The participants for treatment interventions will be the women with mild perineal tears.

#### Study assessment

Any duplicate studies will be excluded. An initial selection of studies identified in the search will be performed using the predetermined inclusion criteria (types of studies, interventions, and participants). Two reviewers will independently assess the titles and abstracts of selected studies. Studies will be excluded if they do not describe the outcomes related to mild perineal tears. Any disagreements will be resolved through discussion after a thorough reading of the paper or by consulting a third researcher.

#### Data extraction

Two reviewers will independently extract the data by reading the full texts. The following data will be extracted from each study if available: study characteristics, outcomes, outcome measurement instruments, and/or definitions provided by the authors for each outcome. If any data are incomplete, the reviewers will contact the studies’ authors by email to obtain the missing data. Before the data are extracted, a consistency evaluation will be conducted between the two reviewers to ensure all analytical details are reliable. Disagreement will be resolved through discussion or by consulting with a third researcher.

#### Data analysis and presentation

Data will be entered into a form, and the outcomes of each study are displayed separately. Then, the outcomes will be classified into different domains (e.g., maternal outcomes and neonatal outcomes) by one of our researchers. Another reviewer will confirm the classification. The number of outcomes used to reflect each domain and the number of different definitions and methods of measurements used will be calculated and presented.

#### Study advisory group

A study advisory group (SAG) will be created, including obstetricians, senior midwives, senior nurses, methodologists, and at least two women with mild perineal tears and one woman with severe perineal tears. The women experiencing perineal tears will be selected from the obstetrics and gynecology clinic. Other members of SAG shall be selected from different regions all over the country. SAG will evaluate the preliminary checklist of the outcome and take part in the consensus meeting to develop the COS.

### Stage 2: Semi-structured interviews

#### The inclusion/exclusion criteria of stakeholders

According to the recommendations of the COS-STAD and COMET handbook (version 1.0) [20, 21], it is necessary to obtain the opinion from stakeholders on mild perineal tear prevention and treatment. Semi-structured interviews will be conducted to acquire stakeholders’ opinions on the outcomes of prevention and treatment of mild perineal tears that should be measured in a clinical trial. This project will facilitate us to understand which outcomes are service users, obstetricians, midwives, and nursing managers focus on and further refine our list of results. The inclusion criteria of service users include pregnant women who are preparing for vaginal delivery and women experiencing or with a history of perineal tears. The inclusion criteria of obstetricians, midwives, and nursing managers include a bachelor’s degree and work for more than 2 years, engaged in the prevention and treatment of perineal injury.

#### Data collection and analysis

The analysis of the data will be conducted simultaneously with the data collection. Investigators will explain the purpose of this study to participants, and they can withdraw at any time. A face-to-face conversation will be conducted after all informed consents are signed. All participants will review the outcome list generated from the systematic review, and we will use open questions as a topic guide. All the interviews will be audio-recorded, the interview will be conducted until the thematic saturation, and no new outcome is obtained. We will use qualitative analysis software (NVivo 11, QSR International Pty Ltd., Burlington, MA) to import the recordings and analyze them through thematic analysis by the framework method. SAG will identify whether these outcomes are new and judge whether they should be added to the list of candidate outcomes. Participants who have completed the interview will be invited to continue to participate in the Delphi survey.

### Stage 3: Delphi survey

#### Participants

A wide variety of stakeholders, including pregnant women or women experiencing mild perineal tears, midwives, obstetricians, nursing managers, methodologists, and researchers with expertise in perineal care, will be recruited. Each participant will be emailed a letter of invitation outlining the study and the link to the online survey. There are no robust standards for the sample size of the Delphi survey [23]. We will use snowball sampling to expand the scale of stakeholders. The experts will invite colleagues who they think meet the criteria for inclusion in the study. In general, the more participants, the more reliable the group judgment will be [24]. We therefore expect to select at least 30 participants in the first round. Informed consent to participate in the study will be obtained from each participant upon registration for the online Delphi questionnaire. Out of consideration for the response rate, a generic reminder email will be sent to related stakeholders to aid the completion of each round. A unique ID number will be assigned to each participant, corresponding to contact information and their responses for each round of the Delphi survey.

#### Delphi round 1

In round 1 of the Delphi survey, the list of outcomes to be scored will be ordered alphabetically to avoid weighting of outcomes caused by the order in which they are displayed and avoid ranking bias in each group. Each outcome will be measured using a 9-point Likert scale, where 1–3 are “not important,” 4–6 are “important but not critical,” and 7–9 are “critical.” This scale has been recommended by the COMET Initiative for measuring the outcomes [16] and has been widely adopted by other COS development studies [25–27]. In the first round of Delphi questionnaires, participants will be given 2 weeks to complete the survey. They can also put forward additional outcomes and provide an associated score that may be included in the second round of questionnaires. A minder will be sent via email when only 3 days are left.

#### Analysis of round 1

The results of round 1 will be summarized using descriptive statistics, including the proportion of participants scoring for each rating point on the Likert scale. Outcomes meeting the consensus out definition will be excluded from the round 2 questionnaire. The newly suggested outcomes listed will be reviewed by the SAG to decide whether they are representative of new outcomes. Those deemed to represent a new outcome based on this review will be added to the round 2 questionnaire accordingly.

#### Delphi round 2

Participants who respond to round 1 will be forwarded the round 2 questionnaire by email with a link to the online survey. Each participant will be presented with the number of respondents and distribution of scores for each outcome from each different stakeholder group and for all groups combined, plus a reminder of their previous score in round 1. They will be asked to consider responses from the other members and invited to re-score the outcome again from 1 to 9, and then to score any additional outcomes identified in round 1. Participants will be given 2 weeks to complete the survey, with a reminder email being sent 3 days in advance.

#### Analysis of round 2

The results of round 2 will be summarized using descriptive statistics. For each outcome, the number of participants who have scored the outcome and the distribution of scores will be noted. Outcomes meeting the consensus out definition will be excluded from the round 3 questionnaire. A comparison will be conducted between each group and all groups combined. Outcomes whose median is ≥ 4 (by any group) will continue to Delphi round 3.

#### Delphi round 3

Participants who complete rounds 1 and 2 will be invited to complete the round 3 questionnaire by email with a link to the online survey. Again, each participant will be presented with the number of respondents and distribution of scores for each outcome from each group and from all groups combined, plus a reminder of their previous score in round 2. They will be asked to re-score the outcome again from 1 to 9, in light of the insight of all stakeholder groups. Participants will be given 2 weeks to complete the survey, with a reminder email being sent 3 days in advance.

#### Analysis of round 3

The results of round 3 will be summarized using descriptive statistics. For each outcome, the number of participants who have scored the outcome and the distribution of scores will be noted. Each outcome will be classified as “consensus in,” “consensus out,” or “no consensus” using the criteria from Harman et al. [28], as summarized in Table 1, which have previously been prespecified in a COS protocol to reduce researcher bias [28]. The results of this process will be brought forward to the consensus meeting.

**Table 1:** Definitions of consensus*

Classification	Definition
Consensus in	≥ 70% participants scoring as 7–9 and < 15% participants scoring as 1–3
Consensus out	≥ 70% participants scoring as 1–3 and < 15% participants scoring as 7–9
No consensus	Anything else

*Developed according to the Management of Otitis Media with Effusion in Cleft Palate protocol [28]

### Stage4: Consensus meeting

A final consensus will be reached during a half-day consensus meeting, which may involve a mixture of face-to-face and teleconference participation. Participants who have completed all rounds of the Delphi study will be invited. The consensus group will include, at a minimum, two representatives of obstetricians, midwives, obstetric nurses, researchers with expertise in perineal care, and at least two service users (women with mild perineal tears or pregnant women). The results obtained from each round of the Delphi survey will be presented to the participants in advance. The responses from round 3 will be used to structure the final consensus meeting. The main objective of the consensus meeting is to discuss the outcomes about which there was disagreement in round 3 of the Delphi study and to validate and agree on a list of the final outcomes which will constitute the COS. Participants will be asked to re-score all of the “without consensus” outcomes anonymously using the same 9-point Likert scale. Outcomes for which at least 70% are scored 1–3 and at most 15% receive a score of 7–9 will be removed from the final COS. Outcomes for which at least 70% are scored 7–9 and at most 15% are scored 1–3 will be included in the final COS. The organizer of the consensus meeting will ensure that participants attending the meeting are collaborative, cooperative, inclusive, and egalitarian to achieve effective consensus.

## Discussion

There is currently no COS specially for mild perineal tears. Our study seeks to develop one COS in this clinical area, aiming to improve the design and operation of future studies related to assessing the effectiveness of prevention and treatment interventions of mild perineal tears for women with vaginal delivery, keeping them in compliance with international standards and guaranteeing the credibility of their results. We will involve a wide range of participants and use recognized techniques to ensure that the ensuing COS is suitable and well accepted in future research. Outcomes important to key stakeholders, particularly to clinicians and service users will be identified through the development of the COS. We hope that the use of the developed COS will ensure the consistency of important outcomes, reduce the reporting bias, and improve the methodological quality and comparability of future studies related to mild perineal tears and the utility of study results.

## Study status

The study plan is to complete the systematic research of literature by December 2021 and the development of the core outcome set by April 2022.

## Abbreviations

COS: Core outcome set
COMET: Core Outcome Measures in Effectiveness Trials
COS-CM-COS-STAD: Core Outcome Set-STAndards for Development
COSMIN: COnsensus-based Standards for the selection of health Measurement INstruments
